# Oral Pemphigus Vulgaris: A Case Report With Review of Literature

**DOI:** 10.7759/cureus.48839

**Published:** 2023-11-15

**Authors:** Hamad Albagieh, Reem F Alhamid, Alaa S Alharbi

**Affiliations:** 1 Department of Oral Medicine and Diagnostic Sciences, College of Dentistry, King Saud University, Riyadh, SAU; 2 Department of Dentistry, College of Dentistry, King Saud University, Riyadh, SAU

**Keywords:** systemic corticosteroids, oral ulcers, desmoglein, autoimmune bullous disease, pemphigus vulgaris

## Abstract

Pemphigus vulgaris (PV) is a chronic autoimmune bullous disease that is characterized by mucocutaneous blister formation resulting in painful erosions. The autoantibody immunoglobulin (Ig) G directed toward glycoproteins desmoglein (Dsg) 3 and desmoglein 1 is the main underlying mechanism behind PV leading to intraepithelial clefting and bulla formation. Patients usually present with oral ulcers causing severe pain and dysphagia that can be misdiagnosed as erythema multiforme (EM) or viral infections. The diagnostic process requires the correlation between clinical, histopathological, and immunopathological findings. Systemic and/or local corticosteroids are considered the cornerstone therapy of PV cases. This article describes a case of a 42-year-old male patient who presented in the Department of Oral Medicine and Radiology with chronic oral ulcers that were diagnosed with PV and treated using systemic corticosteroids.

## Introduction

Autoimmune bullous diseases are autoantibody-mediated disorders characterized by skin and/or mucous membrane blister formation [1]. These diseases can be further subdivided into pemphigus and pemphigoid based on their level of blistering [2]. Pemphigus is a rare chronic mucocutaneous disease that is characterized by intraepithelial bulla formation due to autoantibodies targeting the proteins of the desmosome-tonofilament complex that holds the keratinocytes together [3]. There are multiple subtypes of pemphigus that have been recognized on the basis of their clinical features and pathophysiology, including pemphigus vulgaris (PV), immunoglobulin (Ig) A pemphigus, pemphigus foliaceus (PF), and paraneoplastic pemphigus (PNP) [4]. PV is the most common variant comprising up to 70% of all cases with a reported incidence of 0.76-16 cases per million per year worldwide, with an increased incidence among Ashkenazi Jews and individuals of Mediterranean origin [4-6]. Both sexes are affected with a slightly higher prevalence among females [7]. Initial clinical manifestations include blisters that rapidly rupture to form painful erosions with the oropharyngeal mucous membranes being the most predominately affected site [8]. The pathophysiology of PV is characterized by IgG autoantibodies directed against desmosomal glycoproteins desmoglein (Dsg) 3 and desmoglein 1 of the cadherin family that is responsible for the intercellular adhesion of the squamous stratified epithelium [3]. This article describes a case of oral PV and its proper management. The case report is followed by a review of the literature on etiology, pathophysiology, and possible clinical findings with the variable therapeutic options available.

## Case presentation

A 42-year-old Saudi male patient presented in the Department of Oral Medicine and Radiology with a two-month history of diffuse ulceration on the oral cavity involving the tongue, floor of the mouth, buccal mucosa, lips, and palate in addition to skin lesions that were found on the dorsal surface of the hand and nasal sill. The patient was a nonsmoker and had no relevant family history or known allergy to any specific drug, food, or chemical substance. Associated dysphagia, dehydration, and severe weight loss were also reported. Medical history revealed that he had been suffering from these lesions for approximately four months. He was on topical corticosteroids for the skin lesions and under 25 mg of systemic prednisolone for the oral ulcers. However, after two months of treatment, relapse and recurrence have occurred.

**Figure 1:**
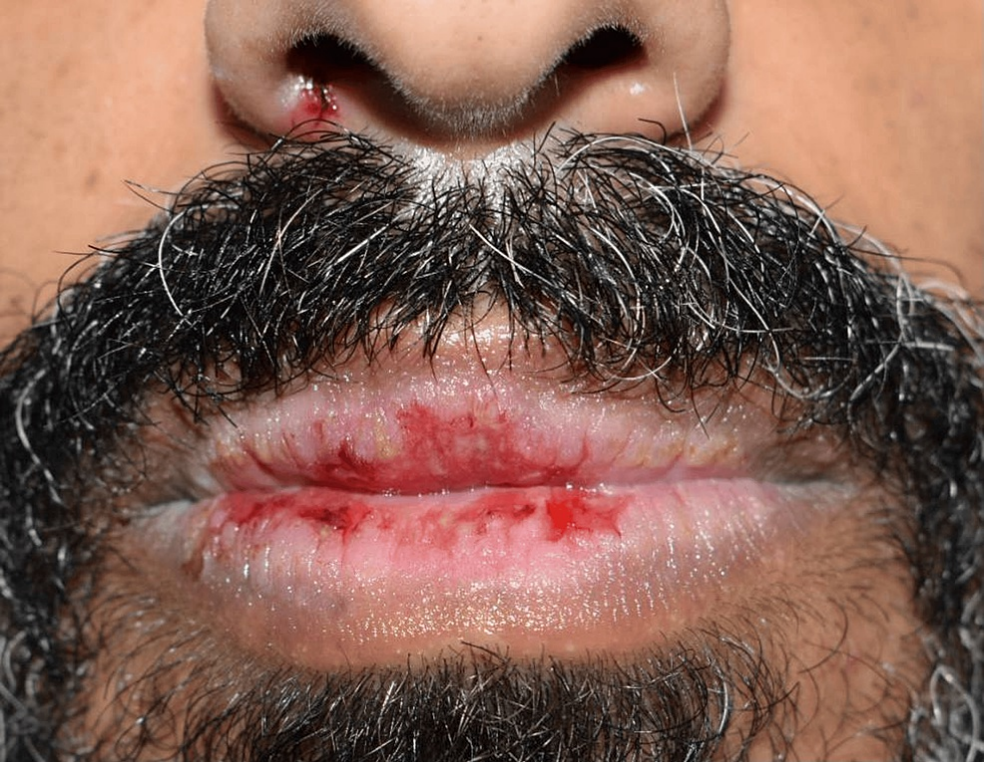
Crusting ulceration on the lips and nasal sill.

**Figure 2:**
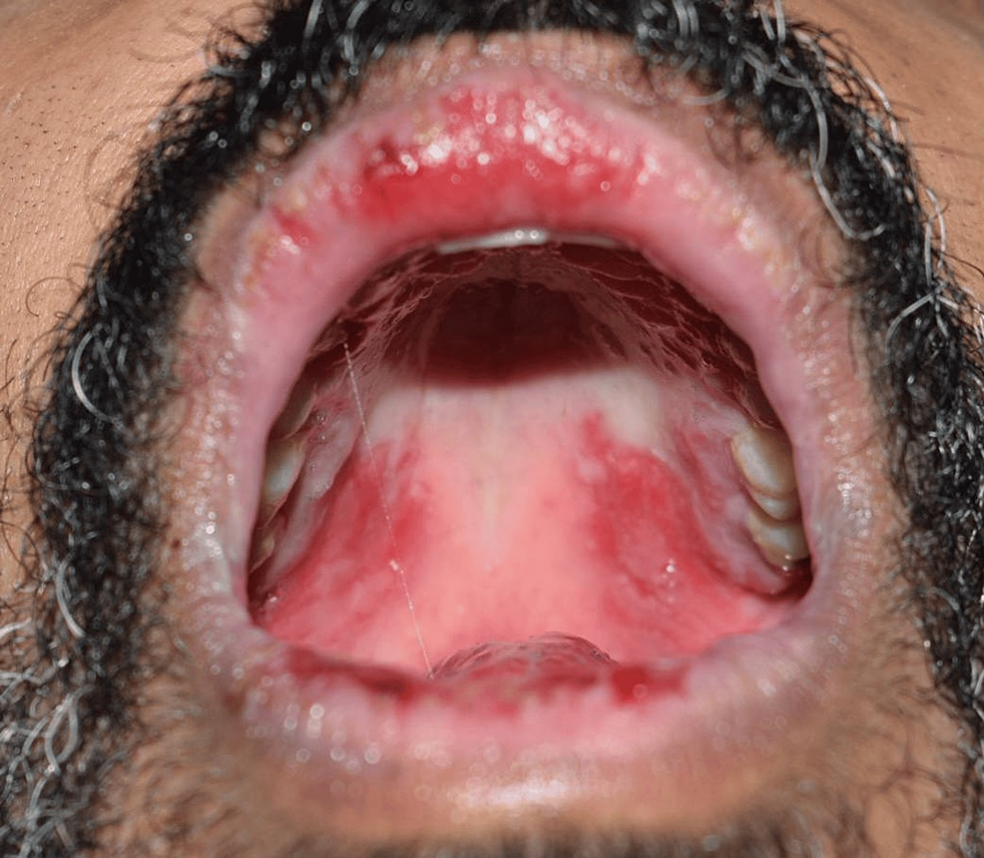
Intraoral diffuse ragged ulcers on the palate.

**Figure 3:**
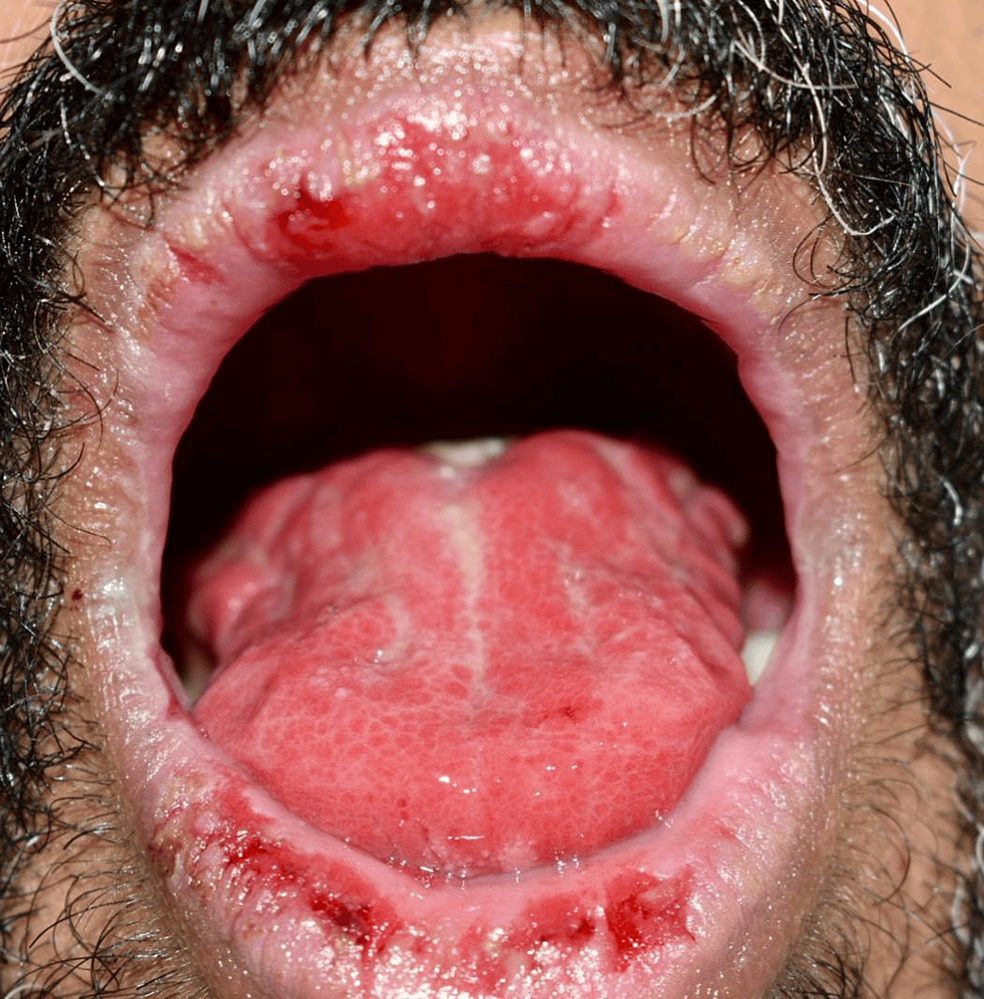
Intraoral diffuse ragged ulcers on the tongue.

**Figure 4:**
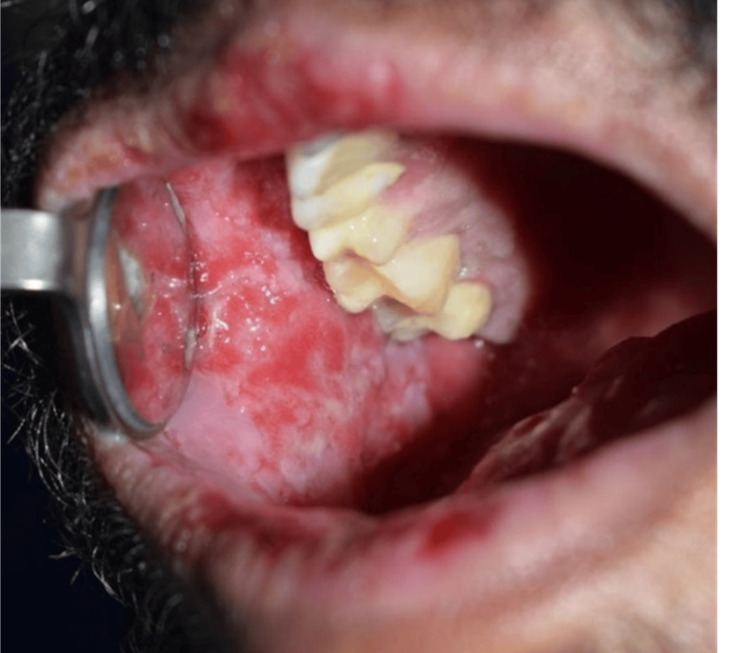
Intraoral desquamative diffuse ragged erosions haphazardly distributed on the right buccal mucosa.

**Figure 5:**
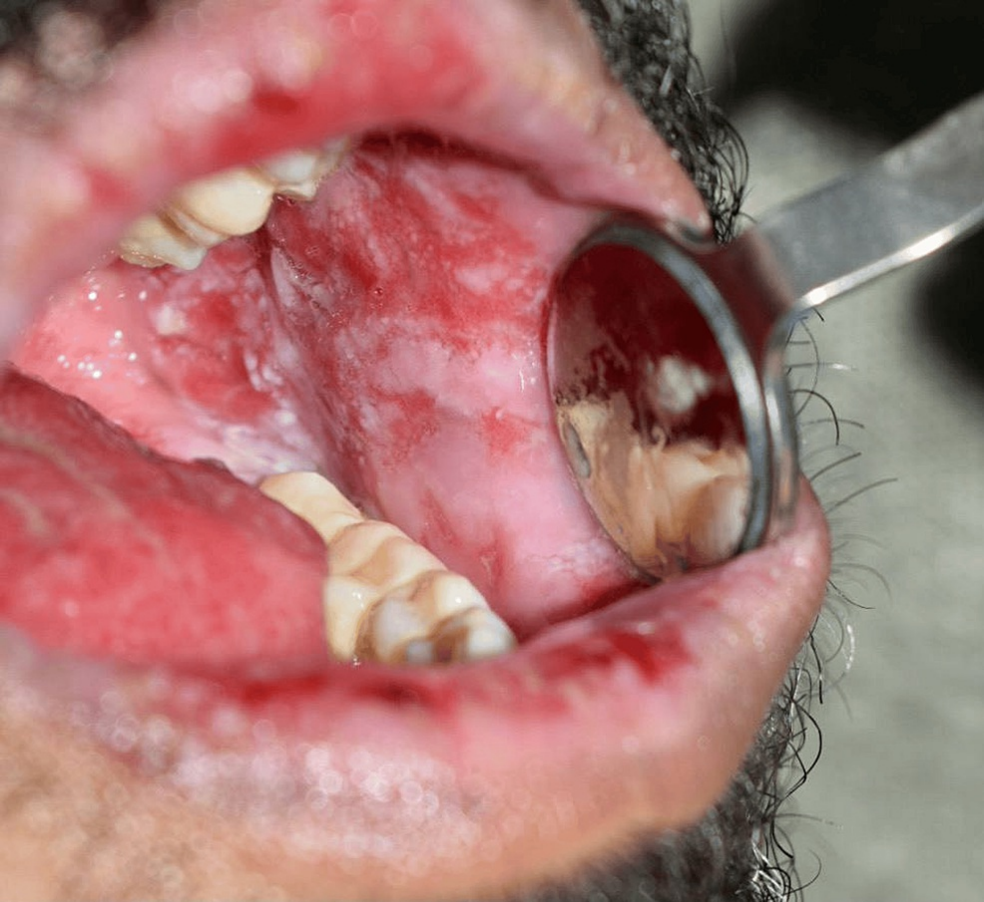
Intraoral desquamative diffuse ragged erosions haphazardly distributed on the left buccal mucosa.

**Figure 6:**
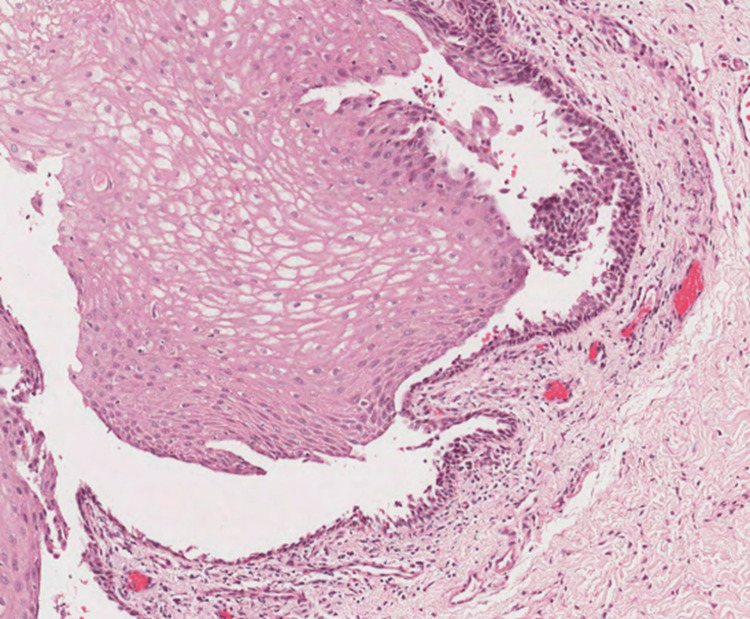
Hematoxylin and eosin (H&E) low-power magnification demonstrating acantholysis and suprabasal clefting with inflammatory infiltrate.

**Figure 7:**
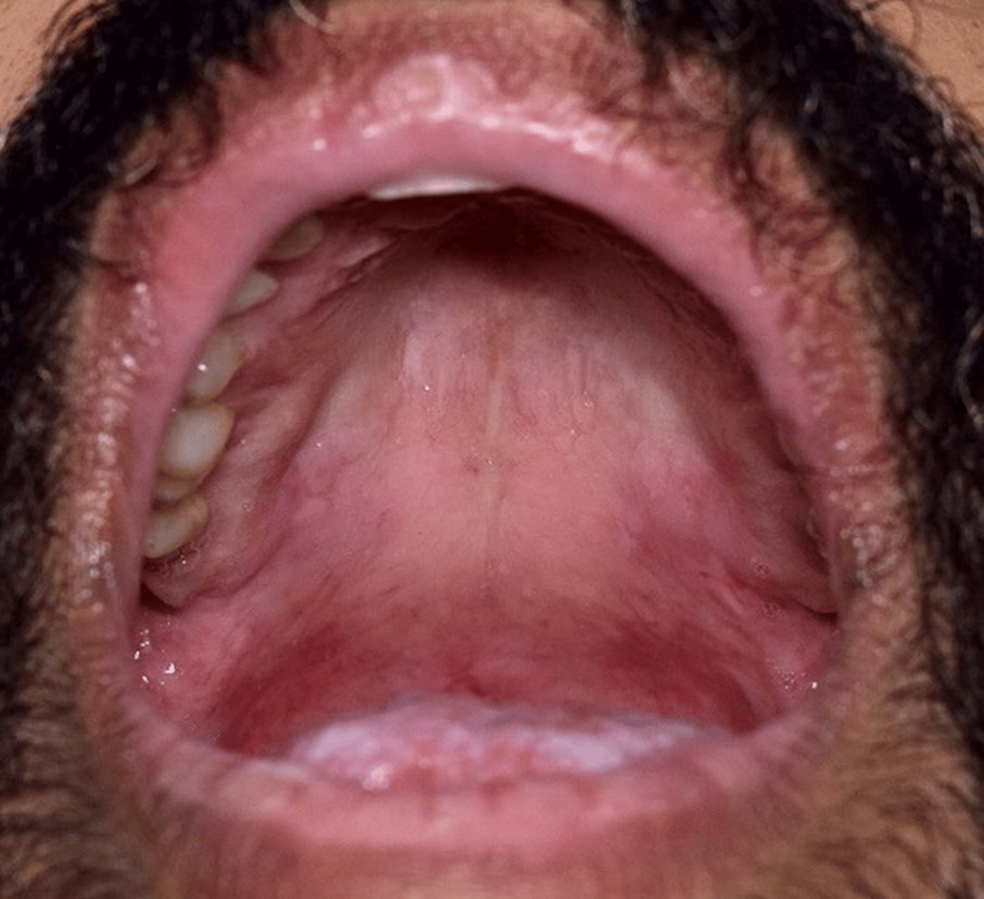
The palate showing healed ulcers without scaring after the use of systemic corticosteroids.

**Figure 8:**
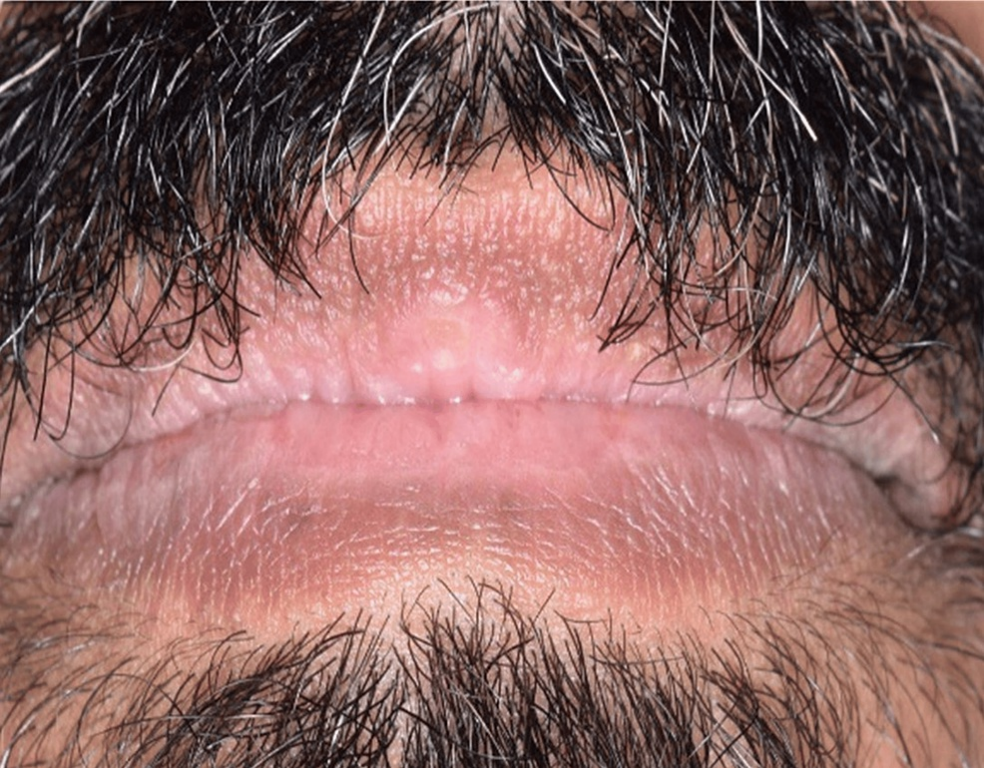
The lips showing healed ulcers without scaring after the use of systemic corticosteroids.

Extraoral examination showed erythema multiforme (EM)-like symptoms including crusting ulceration on the lips and nasal sill (Figure 1). Intraorally, desquamative gingivitis and diffuse ragged erosions were haphazardly distributed on the buccal mucosa, tongue, palate, and floor of the mouth (Figures 2-5). Two perilesional biopsies measuring 0.7 × 0.5 × 0.4 cm were taken from the buccal mucosa and sent for further histopathological and direct immunofluorescence (DIF) examinations. Hematoxylin and eosin-stained sections showed nonkeratinized stratified squamous with acantholysis and suprabasal clefting, leaving one to two layers of basal and parabasal keratinocytes, “row of tombstones” (Figure 6). The superficial lamina propria was mildly infiltrated by mixed inflammatory cells including lymphocytes, plasma cells, and eosinophils, with few mast cells. A final diagnosis of PV was confirmed by DIF showing intercellular immunoglobulin (Ig) G and C3 on a mesh-like pattern, with the C3 being restricted to the basal and suprabasal cell layer. The patient was referred to the Department of Clinical Dermatology with initial treatment of 5 mg prednisone twice daily. After DIF results, a definitive diagnosis of PV was obtained, and 40 mg of systemic prednisone was prescribed along with nystatin antifungal (100000 per 3 mL) mouthwash. A week later, the dose was increased to 60 mg daily with continued use of nystatin mouthwash. After two weeks of the continued use of systemic prednisone, a significant improvement was seen, followed by a period of maintenance where the dose decreased gradually down to 5 mg daily (Figures 7, 8).

## Discussion

Pemphigus vulgaris (PV) is an uncommon chronic autoimmune disease that affects oral and skin tissues in individuals in their fourth and fifth decade of age with slight female predilection [7]. The lesions commonly appear first in the oral cavity as intraepithelial blisters that rupture easily leaving painful erosions in sites subjected to frequent trauma including the buccal mucosa, tongue, palate, and lower lip [8]. Most of the patients come due to oral lesions that cause severe pain, burning sensation, dysphagia, and voice hoarseness [9]. In the present case, the patient came complaining of multiple oral lesions with severe dehydration and dysphagia that resulted in losing approximately 20 kg of weight.

PV possesses a unique ethnic and geographic distribution with a high incidence noted in Ashkenazi Jews and individuals of Mediterranean origin [6]. PV is further subclassified into mucocutaneous-dominant and mucosal-dominant PV. The subclassification is according to the autoantibody profile, desmoglein 1 and desmoglein 3 (Dsg1/Dsg3), which are targeted by PV. The cases with Dsg3+/Dsg1- profile had a mucosal-dominant PV, whereas the cases with Dsg3+/Dsg1+ had mucocutaneous involvement. However, the correlation of mucosal and mucocutaneous involvement with the Dsg profile varies widely between PV cases [10,11].

In this case, the patient presented with signs and symptoms of a similar clinical picture of EM. Hence, a detailed history was essential to distinguish the PV lesions from those caused by acute viral infections including herpes, EM, or atypical ulcers seen in untreated immunocompromised patients with recurrent herpes simplex (RHS) infection. However, none of them were positive in this case.

The diagnostic process of PV involves clinical, histopathological, and immunopathological examinations [12]. Routine mucosal biopsy is used to obtain specific studies to evaluate the tissue changes and to identify the pathogenic autoantibodies. The location of biopsy acquisition must be considered in which perilesional tissue should be sampled at a short distance from the mucosa that is clinically involved with the PV [13]. The histopathological findings typically show supra-basilar acantholysis with a row of tombstone appearance [12]. In addition to routine microscopy, DIF studies will demonstrate the predominant intercellular presence of IgG antibodies in conjunction with complement C3 in a characteristic “fishnet” appearance [14].

The initial goal of treatment is to induce the remission of the condition, followed by a period of maintenance utilizing the lowest drug dosage necessary to keep the disease under control while minimizing adverse effects [15]. Systemic and/or local corticosteroids are considered the primary cornerstone therapy of PV [16]. Although the optimal dose has not been validated yet [16,17], the European Dermatology Forum (EDF) and European Academy of Dermatology and Venereology (EADV) recommend the initial dose of 0.5-1.0 mg/kg/day as the first-line treatment. The prolonged use of systemic corticosteroids for a period of more than four months is associated with other complications such as hypertension, diabetes mellitus, and osteoporosis [15]. A combination or the single use of immunosuppressants such as cyclosporins, azathioprine, and methotrexate can be utilized in the presence of contraindications or side effects related to the prolonged use of corticosteroids [18]. A recent novel improvement in the management of PV involves the use of the biologics rituximab and intravenous immunoglobulin (IVIG), which contributed to a good result in the management of refractory PV. Depending on the patient’s response, the dose can be gradually decreased to the minimal therapeutic dose to minimize the associated side effects [16].

## Conclusions

Pemphigus vulgaris is an uncommon chronic autoimmune disease with a slight female predilection. Dental professionals should be aware of the clinical manifestations and symptoms for proper management. The treatment choice is the use of systemic corticosteroids varying in doses based on the severity of the disease. The diagnosis and treatment process are challenging and require multiple diagnostic methods including clinical, histopathological, and direct immunofluorescence examinations with the latter being the main standard of diagnosis.
